# Characterization of the Enzymatic and Biosorption Processes Involved in the Decolorization of Remazol Brilliant Blue R Dye by *Pleurotus ostreatus* Pellets

**DOI:** 10.3390/jof11080572

**Published:** 2025-07-31

**Authors:** Guadalupe L. Daniel-González, Soley B. Nava-Galicia, Analilia Arroyo-Becerra, Miguel Angel Villalobos-López, Gerardo Díaz-Godínez, Martha D. Bibbins-Martínez

**Affiliations:** 1 Centro de Investigación en Biotecnología Aplicada (CIBA), Instituto Politécnico Nacional, Tlaxcala 90700, Mexico; gdanielg2200@alumno.ipn.mx (G.L.D.-G.); snava@ipn.mx (S.B.N.-G.); alarroyo@ipn.mx (A.A.-B.); mvillalobosl@ipn.mx (M.A.V.-L.); 2 Research Center for Biological Sciences, Autonomous University of Tlaxcala, Tlaxcala 90000, Mexico

**Keywords:** *Pleurotus ostreatus*, fungal pellets, dye biodegradation, biosorption, laccase, dye peroxidase, RBBR

## Abstract

Synthetic dyes are highly recalcitrant and are discharged in large volumes in industrial wastewater, which represents a serious environmental pollution problem. Biological methods for dye degradation are a potentially effective option for these synthetic products. In this study, a strain of *Pleurotus ostreatus* was used to evaluate the decolorization of the Remazol Brilliant Blue R (RBBR) dye added to the culture medium in the exponential growth phase of the fungus. The dye removal capacity of live and inactivated pellets by biosorption, as well as the enzymatic degradation of the dye using a cell-free culture broth considered an extracellular extract (EE), were also evaluated. The activity of laccase and dye-decolorizing peroxidase was determined in both the EE and the intrapellet extract (IPE); their values increased in the presence of dye in the culture medium. A decolorization of 98.5% and 98.0% was obtained in the culture broth and by the EE, respectively; biosorption of the dye by the inactivated pellets was 17 mg/g. The results suggest that the decolorization of the dye is primarily enzymatic, although there are also bioadsorption and bioaccumulation of the dye, which is then enzymatically degraded, and could be used as a carbon source.

## 1. Introduction

Synthetic dyes are widely used in various industries [1,2,3], with the complex chemical structure of these compounds making them highly persistent in the environment and resistant to both light and attack by microorganisms [4]. Anthraquinone dyes are the second most important class of non-degradable textile dyes, preceded by azo dyes [5,6,7,8]. Due to their fused aromatic structure, anthraquinone-based dyes are the textile dyes most resistant to degradation [9,10]. The dye used in the present study, Remazol Brilliant Blue R (RBBR), has been extensively used as a model compound in degradation studies conducted with fungi, pertains to the anthraquinone dyes, and is a notable toxic organopollutant [11,12]. White-rot fungi are highly efficient lignocellulose-degrading microorganisms that cause white rot on wood, from which their name is derived [13]. Among them, *Pleurotus ostreatus* is a well-recognized representative with a robust extracellular enzymatic system and strong potential for environmental applications [9,14]. The use of white-rot fungi to decolorize dye wastewater has been extensively studied [11,15,16,17]. Dye removal can be performed with either live or dead biomass, with the selection between the two also dictating the specific removal mechanisms involved [18]. While the decolorization of dye by fungi is mainly attributed to enzymatic degradation, biosorption processes also play an important role [19]. Fungal mycelium pellets are a source of enzymes that are useful for the degradation of organic compounds and have been reported to present superior characteristics to mycelium itself in terms of biosorption. Moreover, these pellets have an adaptive feature that allows them to survive and efficiently decolorize high dye concentrations and other contaminants, be they inorganic or organic [20,21]. On the other hand, the process of biosorption (bioadsorption and/or bioaccumulation) through the fungal cell wall enables the elimination of said dyes, mainly due to the presence of a great variety of functional groups capable of interacting with inorganic and organic contaminants via various chemical forces [21,22,23]. The ability of *Pleurotus ostreatus* to degrade the complex structures of dyes, making them non-toxic, is primarily attributed to their non-specific multi-enzyme oxidative system, which comprises manganese peroxidases (EC 1.11.1.13), versatile peroxidases (EC 1.11.1.16), laccases (EC 1.10.3.2), and dye-decolorizing peroxidases (EC 1.11.1.19) but no lignin peroxidases [5,11,15,24,25,26]. The relative contribution of such enzymes to the dye biodegradation process varies and depends on several culture conditions, such as pH, temperature, and growth stage [16,27,28,29].

However, other mechanisms also contribute to the efficient transformation of RBBR by *Pleurotus ostreatus*, particularly when mycelial pellets are used, mechanisms that are yet to be fully described. The objective of the present study was to evaluate the decolorization of RBBR dye by *Pleurotus ostreatus* pellets in submerged culture, as well as to determine the participation of both biosorption and enzymatic degradation in the decolorization process.

## 2. Materials and Methods

### 2.1. Organism and Growth Conditions

The present research used *Pleurotus ostreatus* obtained from the American Type Culture Collection (ATCC 32783) (Manassas, VA, USA). The strain was grown and maintained in potato dextrose agar (PDA) prepared with an aqueous wheat straw extract at 10% (*w*/*v*).

### 2.2. Submerged Culture Conditions and Obtaining Extracts

The fungus was grown in submerged culture using a culture medium with the following composition (g/L): glucose, 10; yeast extract, 5; KH_2_PO_4_, 0.6; MgSO_4_-7H_2_O, 0.5; K_2_HPO_4_, 0.4; CuSO_4_-5H_2_O, 0.25; FeSO_4_-7H_2_O, 0.05; MnSO_4_, 0.05; and ZnSO_4_-7H_2_O, 0.001 [30]. The culture was carried out in 250 mL Erlenmeyer flasks with 50 mL of the culture medium at pH 6.5, with each flask inoculated with three mycelial pellets (4 mm in diameter) obtained with a steel punch from the periphery of *Pleurotus ostreatus* colonies grown on PDA for seven days at 25 °C. All flasks were incubated at 25 °C for 16 days and subjected to orbital shaking at 120 rpm on a rotary shaker (SEV-PRENDO 650M, SEV, Puebla, México). After 240 h of culture, the RBBR (R8001 Sigma-Aldrich, St. Louis, MO, USA) was added to each flask, having been adjusted to 500 ppm. A control culture was performed under the same conditions mentioned above, except for the addition of the dye. Both bioprocesses were performed in triplicate, and in all cases, the samples were taken every 24 h after 48 h of culture. In each sample, biomass in the form of pellets (X) was separated from the supernatant via filtration conducted using Whatman No. 4 filter paper (Cytiva Whatman™, Thermo Fisher Scientific, Waltham, MA, USA) and then quantified in dry weight (g/L). The extracellular extract (EE) was considered the supernatant by the present study, while the intra-pellet extract (IPE) was obtained by cell lysis conducted on the pellets (0.25 g/mL on a wet basis, considering 90% humidity for the pellets) with a tissue macerator. For each EE, the pH was measured, and the amount of residual glucose was determined by the 3,5-dinitrosalicylic acid (DNS) method [31], and then the ratio of biomass yield to substrate (Y_X/S_) was calculated.

### 2.3. Decolorization Process and Enzymatic Activity in the Submerged Culture

The decolorization of RBBR obtained in the submerged culture was determined for each EE obtained every 24 h after the addition of the dye by decreasing the absorbance at 595 nm. The decolorization percentage was calculated using Formula (1):

(1) $$\mathrm{Decolorization}\left(\%\right)=\frac{\left(Aa-Ab\right)}{Aa}\times 100$$

where

Aa = Initial absorbance (culture medium with 500 ppm of dye)

Ab = EE absorbance at time t

In both the EE and IPE, laccase and dye-decolorizing peroxidase (DyP) activity was determined using 2,6-dimethoxyphenol (DMP) and 2,2′-azino-bis(3-ethylbenzothiazoline-6-sulfonic acid) (ABTS) as substrates, respectively. Laccase activity was identified by the change in absorbance at 468 nm of the reaction mixture (50 µL of either EE or IPE and 950 µL of 2 mM DMP in 0.1 M phosphate buffer, at pH 6.5) when incubated at 40 °C for 1 min [30]. The activity was reported in international units (U) and defined as the amount of enzyme able to oxidize 1 μmol of substrate per min under the assay conditions (ε = 14,800 M^−1^ cm^−1^). DyP activity was determined using 2.5 mM ABTS in 0.1 M sodium tartrate buffer, at pH 3, as the substrate. The reaction mixture contained 950 µL of substrate, 30 µL of 20 mM H_2_O_2_, and 20 µL of either EE or IPE. Absorbance was read at 436 nm after 1 min of incubation at 45 °C, with the activity reported in U (ε = 29,300 M^−1^ cm^−1^) [32]. For EE and IPE, activity was reported in U/mL and U/g X, respectively.

### 2.4. Dye Adsorption by Inactivated Fungal Pellets

The pellets obtained at 240, 312, and 384 h of the culture generated without dye were inactivated by autoclaving at 121 °C for 15 min, with 50 mL of fresh culture medium and 500 ppm RBBR (25 mg) added, and the mixture was then subject to orbital shaking at 120 rpm and 25 °C for 24 h. Subsequently, the supernatant was recovered by retaining the pellets via filtration, with the absorbance read at 595 nm. A standard RBBR curve of 0–1000 ppm was generated. All experiments and analyses were performed in triplicate and reported as mean ± standard deviation.

### 2.5. Decolorization Using EE

The RBBR, adjusted to 500 ppm, was added to the EEs obtained at 240, 312, and 384 h of the culture without the addition of the dye. The mixtures were incubated at 25 °C for 3 h, with the absorbance read every 30 min from the 1st h of incubation. The percentage of decolorization was obtained using Formula (1).

## 3. Results

### 3.1. Effect of RBBR Dye on Pleurotus ostreatus Growth and Dye Decolorization

**Figure 1:**
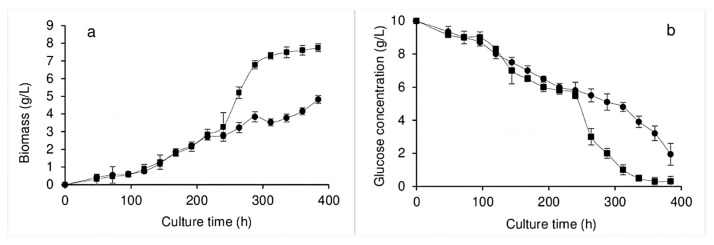
Biomass (**a**) and glucose consumption (**b**) of *Pleurotus ostreatus* grown in submerged culture without dye (●) and with dye (■) added at 240 h of culture.

Figure 1a shows the growth of *Pleurotus ostreatus* in liquid culture, where it can be observed that, in the absence of the dye, exponential fungal growth began at approximately 100 h, while the 384 h that the bioprocess lasted was insufficient to reach the stationary phase. However, the amount of biomass produced increased with the addition of the dye at 240 h, reaching a level approximately 60% higher than that obtained without the dye by the end of the experiment, when the starting point of the stationary phase was also observed. It is important to note that, in both the presence and absence of the dye, the pH of the EE remained relatively constant with minimal variations, ranging from 6.1 to 6.5. This meant that dye protonation or deprotonation that modified its color was not observed, while the pH stability found may have occurred due to the potassium salts and amino acids present in the yeast extract added to the culture medium. The consumption of the carbon source during the bioprocess is shown in Figure 1b. In the culture without dye, a constant glucose consumption without exhaustion was observed (approximately 2 g/L of residual glucose), while, when the dye was added, the glucose consumption increased, achieving near glucose exhaustion by the end of the culture.

**Figure 2:**
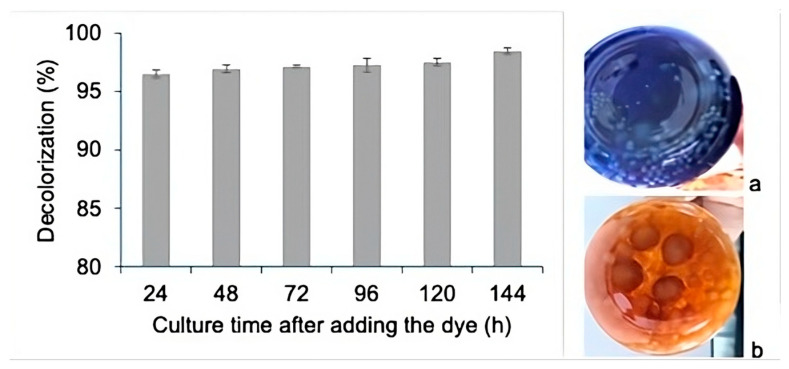
Decolorization percentage of RBBR added at 240 h of submerged culture of *Pleurotus ostreatus.* The images show the bioprocess at the time of addition of the RBBR (**a**) and 144 h later (**b**).

The increased glucose consumption in the presence of the dye is consistent with the increased biomass level produced. These results show that the addition of dye during the growth stage did not inhibit growth but instead promoted the production of biomass. It is important to note that the Y_X/S_ values were found to be 0.59 and 0.80 in the bioprocesses conducted without and with dye, respectively; however, given that these values should be approximately 0.5, the culture without dye can be considered to have presented a congruent value. As the culture carried out with dye presented a value that was 60% above the expected value, this suggests that the dye was used as a carbon source by the fungus of interest. The decolorization process observed by the present study in the *Pleurotus ostreatus* culture was very efficient, wherein 96.5% decolorization was observed when the dye was added at 240 h of growth, just 24 h after the experiment began, with the maximum decolorization value (98.5%) reached 144 h after the dye had been added (Figure 2).

### 3.2. Effect of RBBR Dye on Intra- and Extra-Pellet Enzymatic Activity

**Figure 3:**
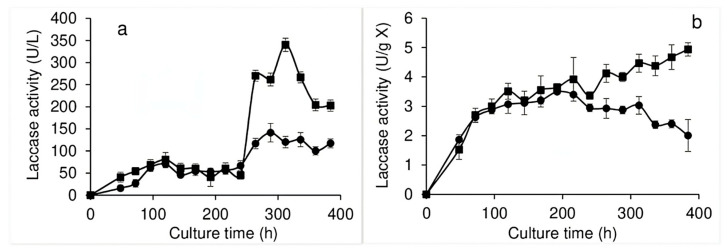
Laccase activity in EE (**a**) and IPE (**b**) of *Pleurotus ostreatus* grown in submerged culture without dye (●) and with dye (■) added at 240 h of culture.

White rot fungi have been observed to be the most efficient fungi for decolorizing different types of dyes, thanks to their ligninolytic enzyme complex, which may include laccases, manganese peroxidases, lignin peroxidases, DyPs, and versatile peroxidases, among others. Given the foregoing, the present study identified laccase and DyP activity in both the EE and IPE. Figure 3a shows the laccase activity of the EE, wherein activity was observed throughout the culture generated without dye, with very low values found during the first 200 h of the culture (approx. 50 U/L). However, during the last 100 h, the values obtained were up to three times higher than those observed before the addition of the dye, while the addition of the dye induced laccase activity values up to seven times higher. The laccase activity observed in the IPE of the culture generated without dye is shown in Figure 3b, which indicates activity throughout the culture, with low values that remained between 2 and 3 U/g X. An increase in activity values was also observed due to the presence of the dye in the culture medium, wherein, by the end of the bioprocess, approximately 2.5 times more activity was obtained than that found in the absence of the dye. The laccase activities observed in the EE and IPE in the culture without dye suggest that there is a basal level of production of these enzymes, in which the dye acted as a strong inducer.

**Figure 4:**
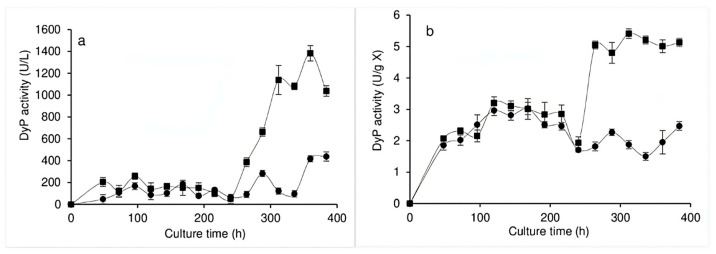
DyP activity in the EE (**a**) and in the IPE (**b**) of *Pleurotus ostreatus* grown in submerged culture without dye (●) and with dye (■) added at 240 h of culture.

The inducing effect of the dye was also observed in the DyP activity, wherein the activity observed in the EE (Figure 4a) revealed that, in the absence of the dye, the DyP activity was approximately 200 U/L throughout the bioprocess, except in the last 48 h, when it doubled. However, 24 h after the addition of the dye, the activity doubled again, and after 120 h, the activity reached values that were seven times higher. Figure 4b shows the DyP activity observed in the IPE and, as with the laccase activity, in the absence of the dye, low values with few fluctuations were observed (2–3 U/g X) throughout the bioprocess, while, when the dye was added, said activity increased by up to three times. It should be noted that, based on the observations and results generated by the present study, during the first 24 h of decolorization, the production of laccase, DyP, and possibly other phenoloxidases increased.

**Figure 5:**
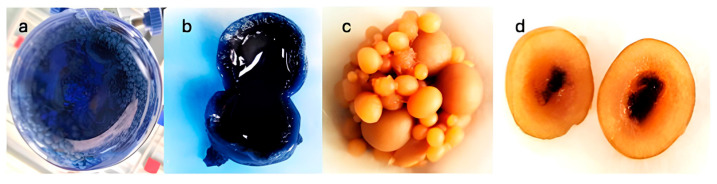
Submerged culture (**a**) and the inside of a pellet (**b**) of *Pleurotus ostreatus* after 24 h of adding RBBR, as well as pellets (**c**) and the inside of a pellet (**d**) after 144 h of adding RBBR.

As shown in Figure 5a, although the culture medium lost a large amount of color, the pellets presented a high amount of color on both their exterior and interior (Figure 5b). However, over time, discoloration of the pellets was observed 144 h after the addition of the dye, as can be seen in Figure 5c,d, which show the degradation of the adsorbed and bioaccumulated dye, respectively.

### 3.3. Dye Adsorption by Inactivated Fungal Pellets

The results obtained with the inactivated fungal pellets showed that the dye presents a high adsorption capacity (Table 1). While the adsorption capacity was found to decrease as the cell aged, given that, as time passed, the amount of biomass produced increased, the total amount of dye adsorbed was also higher. The present study found that the maximum amount of dye adsorbed (almost 17 mg/g X) was observed with the biomass obtained at 240 h, with the amount adsorbed for the biomass obtained at 384 h found to be 18.6% lower. However, the total amount of adsorbed dye was higher with a higher amount of biomass, wherein 23.8 mg of dye was adsorbed with the amount of biomass obtained at 384 h, an amount corresponding to 95.2% of the initial amount applied for each experimental group, namely 25 mg.

### 3.4. Decolorization Using EE

**Figure 6:**
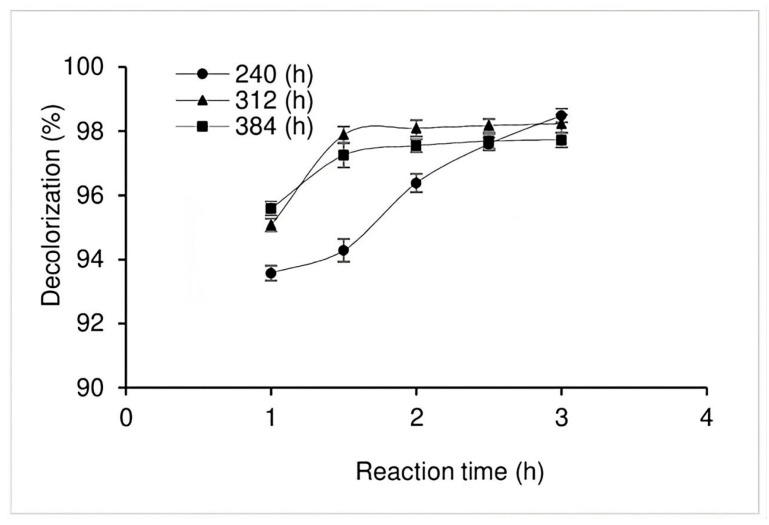
Decolorization of RBBR in the presence of EE’s obtained at 240 (●), 312 (▲), and 384 (■) h.

The decolorization undertaken using EE was also found to be very efficient, with the age of the EE also observed to have had an effect on the percentage of decolorization. As can be observed in Figure 6, after 1 h of contact between the EE and the dye, 93.6, 95.0, and 95.6% decolorization were obtained at 240, 312, and 384 h, respectively, while, after 3 h, the three treatments showed very similar values of approximately 98% decolorization.

## 4. Discussion

The results of the present study show that the addition of RBBR dye during the *Pleurotus ostreatus* exponential growth phase did not inhibit growth and, instead, promoted the production of biomass. By the end of the experiment, production was approximately 60% higher than that obtained without dye, corresponding to 4.8 g/L vs. 7.7 g/L, respectively, while a maximum decolorization value of 98.5% was observed. Although fungal growth provides the basis for the efficiency of the decolorization process of interest, given that both the amount of enzyme secreted and the sorption capacity of the dye depend on the amount of biomass produced, few studies in the literature report the fungal decolorization of dyes in the presence of fungal growth. Furthermore, the Y_X/S_ values give an indication that the dye was being used as a carbon source. One study, which applied the same culture conditions and used the same fungal strain as that of interest to the present study but which also added 500 ppm RBBR at the point of inoculation, obtained an adaptation phase of almost 200 h and a maximum biomass value of 5.73 g/L, while the results of the present study show a maximum biomass value of 7.7 g/L. It should be noted that, in said study, the stationary phase began after 16 days of culture, and the bioprocess was completed after 23 days [33]. Another study grew *Bjerkandera adusta* in three different liquid culture media, one prepared with 2.5 g/L glucose, another with 100 ppm RBBR, and a third containing glucose and RBBR (at the same concentrations as the other two media used). After 30 days of culture, the medium comprising only glucose obtained 2.24 g/L of biomass, while the medium with only RBBR obtained a minimal amount (0.18 g/L), and the medium containing both components obtained 4.46% more biomass than that obtained by the solely glucose medium [34]. Based on the above, it was thought better to add the dye in the exponential phase, as this increases the amount of biomass produced. This may be because, in its exponential phase, the fungus is more metabolically active and has the capacity to secrete the enzymes required to degrade the dye and, moreover, because the existing biomass participates in the biosorption processes, which also degrades the dye.

Since white-rot basidiomycetes are responsible for mineralization of lignin, they have been expected to possess a unique metabolic system for degradation of a variety of aromatic compounds. Different reports indicate that the addition of exogenous aromatic compounds such as dyes to white-rot basidiomycetes growing on glucose as a carbon source has been shown to induce the production of glycolytic enzymes within other enzymes involved in sugar metabolism, thus resulting in the activation of glucose consumption and an increase in growth rate [35,36].

The RBBR decolorization observed in the present study was found to be more efficient and more effective than that reported by other studies, which used lower concentrations of dye to achieve lower percentages of decolorization over a longer period of time. In one such study, two strains of the *Trametes* genus were grown in liquid medium with 10 g/L of glucose, to which 200 ppm of RBBR was added after 24 h of culture. Said study found that both strains decolorized up to 95% of the dye after eight days of culture, although one strain achieved approximately 77% decolorization in only 48 h and the other required four days to decolorize 60% of the dye. In both cases, an increase in decolorization speed was reported to have occurred due to the presence of copper in the culture medium [37]. Another study grew *Bjerkandera adusta* in a liquid medium supplemented with 2.5 g/L glucose, to which 100 ppm RBBR was added at the point of inoculation. After seven days of culture, 20% discoloration was observed, with the maximum discoloration of 95% obtained after 18 days [34]. Grandes-Blanco et al. [33] used the same culture medium and strain applied by the present study to evaluate the decolorization of 500 ppm RBBR, which was added at the point of inoculation. While the authors of said study observed only 10% decolorization after four days of culture, respective decolorization levels of 75, 90, and 95% after seven, ten, and 14 days, respectively, were obtained. Another study grew a *Pleurotus ostreatus* strain in liquid medium, to which 2 g/L of glucose was first added and then 200 ppm of RBBR, after three days of culture. After 24 h, a near 80% decolorization level was achieved, with maximum decolorization (95%) obtained three days after the addition of the dye [38]. A *Pleurotus ostreatus* strain was grown in potato dextrose broth in the presence of 50 ppm RBBR, achieving, by the second day of cultivation, a maximum decolorization of 70%, which remained stable until Day 5 of the study, when the bioprocess was stopped [15]. A recent study isolated 70 fungal strains from dye wastewater obtained from the textile industry, of which strains 19 decolorized RBBR, and strains SN8c and SN40b obtained the highest decolorization levels. Both strains, grown in liquid medium with 1 g/L glucose and 400 ppm RBBR, achieved decolorization levels of 91.3 and 84.5%, respectively. The above-mentioned study observed the most favorable decolorization conditions at a pH ranging from three to five [39].

With regard to the effect of RBBR dye on intra- and extra-pellet enzymatic activity, it is worth mentioning that many studies have found the decolorization of textile dyes to be mainly related to the extracellular activity presented by reducing enzymes such as azoreductases and/or oxidase enzymes, mainly laccases but also peroxidases [40]. The present study determined laccase and DyP activity in both the EE and the IPE during the bioprocess undertaken without dye, finding that, after the addition of RBBR to the culture medium, both enzymatic activities increased in both extracts. Studies have determined the activity of fungal enzymes during the decolorization process, with, for example, two strains of the *Trametes* genus with the ability to decolorize RBBR (200 ppm in liquid culture) producing laccase enzymes. The addition of copper to the culture medium resulted in an increase in the activity of these laccase enzymes, although the dye was also found to act as an inducer. It should be noted that, although the maximum decolorization of RBBR was not dependent on the presence of copper, it did accelerate the process, possibly due to the higher level of laccase activity induced by both compounds [37]. Another study found that the decolorization of RBBR by a *Bjerkandera adusta* strain in liquid culture correlated with both a decrease in the levels of certain phenolic compounds in the supernatant (suggesting the biodegradation of the dye) and the maximum level of peroxidase enzyme activity [34]. Various studies have evaluated the decolorization of RBBR dye by *Pleurotus ostreatus* in liquid culture, observing a strong relationship between oxidase enzyme activity and decolorization. The decolorization process began in the first days of the culture, suggesting that the decolorization process requires the participation of all ligninolytic enzymes and that the addition of inducers of these enzymes accelerated decolorization, which reached almost 100% in 24 h [33,38]. Another study evaluated laccase, manganese peroxidase, and lignin peroxidase activity in three fungal strains (*Trametes hirsuta*, *Ceriporia* sp., and *Cymatoderma dendriticum*) grown for nine days under static conditions. While laccase activity was observed in the cultures of the three fungi, the other two enzymes presented minimal levels; however, laccase activity increased in the presence of guaiacol in all three fungi of interest. The highest level of laccase activity was found for *Trametes hirsuta,* which was able to decolorize up to 65% of the RBBR in 72 h [41]. Bernal et al. [42] isolated filamentous fungi from textile industry wastewater, evaluating their ability to produce laccase, cellulase, amylase, and lipase enzymes and determining their potential for decolorizing RBBR dye. An *Aspergillus sydowii* strain presented cellulase, amylase, and laccase activities, showing the ability to decolorize 74.7% of RBBR in liquid medium. The mycelium of *Trametes hirsuta* immobilized on nylon sponges showed a high level of decolorization (95.39%) of mixed textile dyes (Navy EC-R, Ruby S3B, and Super Black G) in real industrial wastewater samples, with the main dye-decolorization mechanism found to be biodegradation. While manganese peroxidase and laccase were found to be the most prevalent enzymes, it should be noted that the enzyme lignin peroxidase was not detected [43].

Many reports suggest that the process for decolorizing RBBR is enzymatic. However, it has also been suggested that adsorption may play an important role in the overall process, as the adsorption of the dye into the fungal mycelium may allow the chromophores to come into contact with the degradative enzymes associated with the cell surface [40,44]. Fungal mycelium pellets are also a source of enzymes useful for the degradation of organic compounds and have been reported to present superior characteristics than mycelium itself in terms of biosorption due to their porous structure and high surface area. Moreover, these pellets have an adaptive feature that allows them to survive and efficiently decolorize high dye concentrations and other contaminants, be they inorganic or organic [21]. It should be noted that, based on the observations and results generated by the present study, during the first 24 h of decolorization, the production of laccase, DyP, and possibly other phenoloxidases increases. However, the processes involving the enzymatic degradation, adsorption, and bioaccumulation of the dye were carried out simultaneously. As shown in Figure 5a, although the culture medium lost a large amount of color, the pellets presented a high amount of color on both their exterior and interior (Figure 5b). However, discoloration of the pellets was observed 144 h after the addition of the dye, as can be seen in Figure 5c,d, which show the degradation of the adsorbed and bioaccumulated dye, respectively.

It should be noted that, while the biosorption of synthetic dyes can be carried out with either living or dead biomass, some authors suggest that biosorption conducted with dead biomass could be a simpler process [27]. However, based on that observed by the present study, the dye adsorption and bioaccumulation processes, when conducted with living biomass, presented subsequent enzymatic degradation, which, thus, improves the bioremediation process. The dye adsorption results obtained by the present study using heat inactivated fungal pellets showed a high dye-adsorption capacity (a maximum of 17 mg/g), with both a direct relationship with the amount of biomass and a negative impact of pellet age observed; it is worth mentioning that these results only show the existence of bioadsorption of the dye, so it should be considered that this process by living biomass in the submerged culture may be less efficient than that of biomass inactivated by autoclave sterilization, which can be attributed to the fact that this type of inactivation causes greater exposure of the functional groups (carboxyl, amino, thiol, sulfhydryl, phosphate, etc.) available as sorption sites on the cell surface, since there may be rupture of the membrane and cell wall of the fungus, increasing the contact area where the polysaccharides, lipoproteins and lipopolysaccharides are located, which are the biomolecules responsible for the binding of the dye molecules [45]. Aksu and Balibek [46] studied the biosorption of the anionic dye Yellow RL by dead fungal biomass taken from *Rhizopus arrhizus*, obtaining a biosorption capacity of 85.4 mg/g for the use of dead dry biomass on 100 mg/L of dye. Another study used 20 mg of dead *Aspergillus fumigatus* biomass (previously dried and pulverized) to determine its biosorption capacity when applied to 10 mL of methylene blue dye at 12 mg/L. Said study found a biosorption level of 68% over 120 min at a neutral pH, although the maximum biosorption obtained was 93.5% at an alkaline pH, leading to the conclusion that the biosorption observed was related to the amount and surface area of the biosorbent [47]. A previous study inactivated, via sterilization, the biomass of a *Pleurotus ostreatus* strain grown in liquid culture in order to then evaluate its ability to adsorb a mixture of triphenylmethane brilliant green and Evans azo blue dyes, achieving a 37% adsorption level; however, when applied with live biomass, this methodology achieved 79.2% decolorization [48]. Dogan et al. [49] evaluated the biosorption capacity of the dried pulverized biomass of a *Pleurotus ostreatus* strain when applied on indigo-carmine dye via the use of 0.1, 0.2, and 0.5 g of dead biomass on 50 mg/L of the dye, finding that, after 120 min, approximately 60, 90, and 75% removal, respectively, was obtained. However, the authors also found that increasing the concentration of the dye from 100 mg/L to 500 mg/L reduced the removal level from approximately 80% to 60%. Thampraphaphon et al. [43] used dead and live cells of a *Trametes hirsuta* strain that had been grown immobilized on nylon sponge to evaluate the adsorption of mixed textile dyes (Navy EC-R, Ruby S3B, and Super Black G) from real industrial wastewater samples. Under optimal conditions, said study found that 28.98% of the dye was removed by the dead cells. However, the immobilized live fungal cells achieved 89.21% decolorization, suggesting that biodegradation is responsible for the 60.23% difference in decolorization level, namely that it is the main process involved in the decolorization. It should also be noted that the nylon sponge adsorbed only 4.04% of the dye.

Additionally, the present study evaluated decolorization efficiency using EE from different *P. ostreatus* culture times, observing high RBBR decolorization percentages, 93.5 to 98.0%, at 240 h and 384 h, respectively. Shin and Kim [50] purified an extracellular peroxidase enzyme from the culture medium of *Pleurotus ostreatus*, observing that it was able to decolorize RBBR. The peroxidase had a molecular mass of 71 kDa, as measured using SDS-PAGE, while its respective optimal pH and temperature values were 3.0–3.5 and 25 °C. The Km of the enzyme, when used with the RBBR, was 10.99 mM, with the enzyme also presenting affinity with several phenolic compounds and artificial dyes. Other authors obtained extracts both from the substrate colonized by *Pleurotus ostreatus* at 20, 25, and 28 days of culture and from the substrate after harvest, determining the capacity of the extracts to degrade RBBR. In addition, the enzymatic activities of peroxidase and laccase were determined, wherein, for all extracts, laccase activity was minimal, and peroxidase activity was identified in those extracts taken from the colonized substrate. The highest peroxidase activity and the highest percentage of discoloration were both obtained for the extract obtained from the 25-day culture. It is worth mentioning that neither peroxidase activity nor discoloration was observed in the spent extract [12]. Teixeira et al. [51] compared a commercial laccase taken from *Aspergillus oryzae* and an extract obtained from *Pleurotus ostreatus* culture residues in terms of their effectiveness for decolorizing the reactive dyes Drimaren Blue X-3LR (DMBLR), Drimaren Blue X-BLN (DMBBLN), Drimaren Rubinol X-3LR (DMR), and Drimaren Blue C-R (RBBR). The decolorization metrics considered for both laccases were dye concentration and reaction time, while the addition of ABTS was considered as a mediator. The ABTS was necessary for the decolorization of the DMR (80–90%, 1 h) and RBBR (80–90%, 24 h) using both laccases, while it was not required for the decolorization of either the DMBLR (85–97% in 1 h) or the DMBBLN. In 24 h, the commercial laccase obtained 60% decolorization and the crude extract 80%. Another study produced a laccase from *Lentinus crinitus* grown on agro-industrial waste and tested it for RBBR decolorization, using coffee peel or citrus pulp pellet culture media and different nitrogen sources. The highest laccase activity was obtained for the coffee peel medium, with maximum decolorization values obtained in 24 h and 74% and 76% obtained using the enzymatic extracts of citrus pulp pellet and coffee peel, respectively [52]. In another study, the decolorization of the Remazol Brilliant Violet 5R dye by a purified laccase produced by a *Pleurotus ostreatus* strain was optimized to include pH, temperature, incubation time, agitation, dye concentration, and enzyme concentration. The maximum decolorization obtained was 95.72% at pH 6.0, a temperature of 40 °C, 60 min of reaction with agitation at 50 rpm, and the application of 50 ppm dye and 100 U/mL enzyme [53].

Another study used enzymatic extracts obtained from the fungi *Coriolus versicolor* and *Pleurotus ostreatus* to decolorize RBBR, with both fungi grown in solid-state fermentation using wheat bran and soybean residues as support. After 17 days, the enzymatic extracts were obtained from the substrates in the proximity of the fungi as it grew. The best conditions for the decolorization of RBBR by both extracts were found to be a pH of 4.0, a temperature of 30 °C, and 20 U of laccase activity, while 100 mg/L and 50 mg/L of dye were used for *Coriolus versicolor* and *Pleurotus ostreatus*, respectively. Under the above conditions, decolorization values of 80.42% and 70.42% were obtained for *Coriolus versicolor* and *Pleurotus ostreatus*, respectively. Higher laccase activity was observed in the *Coriolus versicolor* culture than the *Pleurotus ostreatus* culture [54].

## 5. Conclusions

*Pleurotus ostreatus* showed the ability to decolorize RBBR dye in liquid culture through enzymatic degradation, biosorption, and bioaccumulation processes. However, these processes did not occur sequentially or cumulatively; rather, they may have acted simultaneously and synergistically. Furthermore, given the decolorization of the culture broth and the biomass that adsorbed and accumulated the dye, it is suggested that enzymatic degradation was the main mechanism. It was also observed that laccase and DyP activities, both in the extracellular and intrapellet extracts, were higher in the presence of the dye, although other peroxidase enzymes may be involved in dye degradation. The results obtained by the present study show the potential of *Pleurotus ostreatus* for RBBR decolorization in a short time, and it is suggested that the fungus degrades the dye and uses it as a carbon source.

## Figures and Tables

**Table 1:** Dye adsorbed by inactivated fungal pellets.

Pellet Age (h)	* Biomass (g X)	* Amount of Adsorbed Dye (mg)	^+^ Dye Adsorbed (mg/g X; %)	Inactivated Pellets with Adsorbed Dye
240	1.08 ± 0.05	18.05 ± 0.74	16.72; 72.2	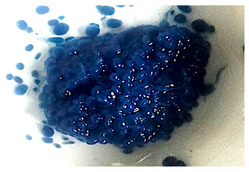
312	1.45 ± 0.08	22.09 ± 0.23	15.24; 88.4	
384	1.75 ± 0.05	23.80 ± 0.02	13.60; 95.2	

* Values are the mean ± standard deviation of three replicates. ^+^ Calculated from the values in the previous columns and based on the initial amount of dye (25 mg).

## Data Availability

The original contributions presented in the study are included in the article; further inquiries can be directed to the corresponding authors.
